# Improving end-of-life care for people with dementia: a mixed-methods study

**DOI:** 10.1186/s12904-023-01335-w

**Published:** 2024-01-30

**Authors:** Zoi Triandafilidis, Sally Carr, Daneill Davis, Sarah Yeun-Sim Jeong, Jacinta Hensby, Daniel Wong, John Attia, Nicholas Goodwin

**Affiliations:** 1 Central Coast Research Institute, Gosford, NSW Australia; 2 Central Coast Local Health District, Gosford, NSW Australia; 3 University of Newcastle, Newcastle, NSW Australia; 4 The University of Sydney, Sydney, NSW Australia; 5 Hunter Medical Research Institute, Newcastle, NSW Australia

**Keywords:** Palliative care, End of life, Dementia, Carers, Healthcare professionals, Mixed-methods, Needs-based care, Person-centred, Death & dying, Care coordination

## Abstract

**Background:**

Improving palliative and end-of-life care for people with dementia is a growing priority globally. This study aimed to integrate multiple perspectives on end-of-life care for people with dementia and carers, to identify clinically relevant areas for improvement.

**Methods:**

The mixed-methods study involved surveys, interviews, and workshops with two participant groups: healthcare professionals and carers (individuals who provided care and support to a family member or friend). Healthcare professionals were invited to complete an online adapted version of the Australian Commission on Safety and Quality in Health Care, End-of-Life Care Toolkit: Clinician Survey Questions. Carers completed a hard copy or online adapted version of the Views of Informal Carers—Evaluation of Services (Short form) (VOICES-SF) questionnaire. Interview schedules were semi-structured, and workshops followed a co-design format. Findings were integrated narratively using a weaving approach.

**Results:**

Five areas in which we can improve care for people with dementia at the end of life, were identified: 1) Timely recognition of end of life; 2) Conversations about palliative care and end of life; 3) Information and support for people with dementia and carers; 4) Person-and-carer-centred care; 5) Accessing quality, coordinated care.

**Conclusions:**

There are multiple areas where we can improve the quality of end-of-life care people with dementia receive. The findings demonstrate that the heterogeneous and challenging experiences of living with and caring for people with dementia necessitate a multidisciplinary, multifaceted approach to end-of-life care. The identified solutions, including care coordination, can guide local development of co-designed models of end-of-life care for people with dementia.

**Supplementary Information:**

The online version contains supplementary material available at 10.1186/s12904-023-01335-w.

## Background

Dementia is the seventh leading cause of death globally [1], and a leading cause of death for people in Australia, with 9.6% of all deaths in 2020 attributed to dementia [2]. The number of people living with dementia is expected to more than double by the year 2058 [2]. For life-limiting illnesses such as dementia, palliative care is recommended [3]. The World Health Organisation defines palliative care as “an approach that improves the quality of life of people facing problems associated with life-threatening illness” [4]. End-of-life care is typically provided when a person is considered to be in their last 12 months of life and refers to necessary care focussed on individual needs and preferences [5]. A palliative approach to care for people with dementia at the end of life has many benefits for the individual [3] and may also be cost saving [6]. However, people with dementia, both in Australia and internationally, do not always get appropriate access to palliative and end-of-life care [7, 8]. From July to December 2022 only 5.1% of people referred to specialist palliative care services in Australia had a dementia diagnosis [9].

There are known barriers preventing timely access to palliative and end-of-life care for people with dementia. Firstly, dementia is often not considered to be a terminal illness by healthcare professionals, people with dementia, or their families [10]. Secondly, accurate prognostication due to the variable trajectory of the illness is a key barrier [11], making entry into palliative care programs at the appropriate time, difficult. van Riet Paap and colleagues found that professionals often have different opinions regarding the time point when to consider a person with dementia in need of palliative care [12]. Thirdly, palliative care programs or services are also often ill-equipped to support the agitation experienced by many people with dementia in their last week of life [13]. These structural barriers prevent healthcare professionals from providing the care they feel is required [14]. While the need to address these barriers and to improve access to quality end-of-life and palliative care for people with dementia is well established [15], existing literature has called for more clinically relevant evidence and collaborative approaches to establish the key components of palliative and end-of-life care for people living with dementia and their carers that can be translated into practice. Previous studies have mostly focussed on the individual perspectives of healthcare professionals [14], or carers [16]. Therefore, the aim of this study was to integrate multiple perspectives on care provided at the end of life for people with dementia and carers, to identify clinically relevant areas for improvement.

## Methods

A mixed-methods study was designed with the aim of identifying areas for improving care for people with dementia at the end of life. The project was clinician-led to establish clinically relevant evidence to inform practice, as well as the co-design of a new model of care for people with advanced dementia. The perspectives of English-speaking healthcare professionals and carers of people who died with dementia were gathered using existing survey instruments, interviews, and workshops. Within-method (two different surveys designed for different participant groups) and between-methods (surveys, interviews, and workshops) triangulation were used to maximise the range of perspectives on end-of-life care and overcome the limitations of a single method [17]. Descriptive statistics were produced from the surveys, and interviews and workshops were analysed thematically. Integration of the six datasets occurred at the interpretation and reporting level using narrative weaving. Ethics approval for this project was granted by the Hunter New England Human Research Ethics Committee (2021/ETH11309).

### Participants

Participants gave informed consent to participate after receiving written and verbal information. Participation was voluntary, and the participants could withdraw at any time without consequences.

### Healthcare professionals

People currently working as healthcare professionals providing care for people with dementia on the Central Coast (a region of New South Wales, Australia), were invited to participate in a survey, an interview, or both. Upon completion of the survey and/or interview, participants were invited to the workshop. A total of 66 healthcare professionals responded to the online survey, 10 participated in an interview, and 12 in the workshop. Most survey participants were female (83.3%), nurses (56.0%) and allied health professionals (28.8%), working predominantly in public hospitals (72.7%), and had been providing care for people with dementia for 10 years or more (66.7%) (Table 1).

**Table 1:** Healthcare professional demographics

Variable	Survey (*n* = 66) N (%)	Interviews (*n* = 10) N
**Gender**		
Man / Male	10 (15.2)	3
Woman / Female	55 (83.3)	7
Non-binary	1 (1.5)	0
**Clinician cohort**		
Consultant	5 (7.6)	0
GP	0 (0)	0
Junior doctor	2 (3.0)	0
Nurse	37 (56.0)	8
Allied health professional	19 (28.8)	2
Care worker	1 (1.5)	0
Community case manager	1 (1.5)	0
Other ^a^	1 (1.5)	0
**Years of clinical experience**		
0–1 year	2 (3.0)	0
2–5 years	10 (15.2)	0
6–10 years	10 (15.2)	2
> 10 years	44 (66.7)	8
Place where majority of care given*		
Hospital	48 (72.7)	7
Person’s private home	18 (27.3)	3
Residential aged care	12 (18.2)	2
Other	7 (10.6)	0

^*^Multiple choice question, proportion of respondents who checked the option reported

^a^ Clinical pharmacist

### Carers

Carers were identified through a linked audit of Central Coast Local Health District electronic medical records in March 2022. The audit identified the primary contact of people who died with a diagnosis of dementia between January 2015 and December 2019, inclusive, and had accessed services from the Central Coast Local Health District. We selected a 5-year period to capture the sample size required for the linked audit. A pre-COVID period was selected as healthcare service delivery was altered from 2020 onwards. A total of 598 carers were identified as eligible for participation and were invited to participate in a survey, an interview, or both. Upon completion of the survey and/or interview, participants were invited to the workshop. We received 57 responses (53 hard-copy surveys and four were completed online) (response rate of 9.5%). Eleven carers participated in an interview, and two in the workshop. Most survey participants were female (59.7%), more than a third were aged 60–69 years (36.8%), and most were the child of the deceased (59.7%) (Table 2).

**Table 2:** Carer demographics

Variable	Survey (*n* = 57) N (%)	Interviews (*n* = 11) N
**Age (years)**		
< 50	2 (3.5)	0
50–59	11 (19.3)	2
60–69	21 (36.8)	5
70–79	17 (29.8)	2
80–89	6 (10.5)	2
**Gender**		
Man / Male	23 (40.4)	5
Woman / Female	34 (59.7)	6
**Relationship to care recipient**		
Spouse	12 (21.1)	2
Child	34 (59.7)	8
Sibling	3 (5.3)	0
Child-in-law	4 (7.0)	0
Parent	3 (5.3)	0
Friend or neighbour	1 (1.8)	1

### Procedure

#### Survey

The healthcare professional survey explored experiences of providing care for people with dementia and their families at the end of life, using Likert-style, multiple-choice, and free-text questions. This survey asked respondents to focus on end-of-life care provided in the final 12 months of life. This survey was adapted with permission from the Australian Commission on Safety and Quality in Health Care, End-of-Life Care Toolkit: Clinician Survey Questions [18] (Supplementary file 1). The survey took approximately 10–15 min to complete and was available through REDCap (Research Electronic Data Capture), a secure, web-based software platform [19]. Participants had a 2 month period in which to return the survey, between 11 March and 16 May 2022. A link to the online survey for healthcare professionals was shared by the local health district, and community organisations. The study was also promoted in newsletters, network and agency meetings, and by key stakeholders.

The survey for carers explored experiences of caring for a person at the end of life who had dementia. This survey asked respondents to focus on end-of-life care provided in the last 3 months of life, the final hospital admission, the last 2 days of life, and circumstances surrounding the death. The survey was adapted with permission from the Views of Informal Carers—Evaluation of Services (Short form) VOICES-SF questionnaire, a verified survey instrument that has been used with populations who have experienced bereavement [20] (Supplementary file 2). The survey used Likert-style, multiple-choice, and free-text questions and took up to 30 min to complete. Carers had the option of completing the survey online using REDCap, or paper copy. Hard copy survey responses were manually entered into REDCap by a member of the research team. Participants had a 1 month period in which to return the survey in a reply-paid envelope during July 2022.

#### Interviews

Seventeen healthcare professionals expressed interest in participating in an interview and were contacted. Seven healthcare professionals were unreachable. Ten interviews were held in April and May 2022. Six interviews were completed online using videoconferencing software, and four were completed by phone. Interviews ranged between 20 and 37 min. A semi-structured interview schedule was created for the study which explored healthcare professionals’ perceptions of end-of-life care for people with dementia in their place of work (Supplementary file 3).

Thirty-three bereaved carers expressed interest in participating in an interview. Fifteen carers were purposively contacted to represent a range of demographic characteristics (including relationship to care recipient, age, gender), and 11 were interviewed during July and August 2022. Nine interviews were completed by phone, and two carers chose to participate in a joint online interview using videoconferencing software. Interviews ranged between 20 and 64 min. A semi-structured interview schedule was created for the study which explored carers’ perceptions of the care provided to their care recipient in their final year of life (Supplementary file 4).

#### Workshops

Healthcare professionals and carers who participated in a survey and/or interview were also invited to attend a workshop to collaboratively review the project findings, provide feedback on the results, and to begin to co-design an end-of-life pathway for people with dementia. The workshops for healthcare professionals and carers were held separately to minimise any perceived differences in status or power between the two groups which may have led to the discussions being dominated by healthcare professionals. The first workshop took place in July 2022 with 12 healthcare professionals, who had backgrounds in social work, occupational therapy, palliative care, and nursing. In August 2022 the second workshop took place with two carers. Workshops were held online due to restrictions relating to the COVID-19 pandemic. Prior to the workshops, participants were sent a summary of the survey and interview findings. The workshops were structured in line with the Agency for Clinical Innovation’s guide for developing a Model of Care [21] which includes identifying and prioritising issues, designing solutions, and implementation and sustainability. The workshops were recorded, and field notes taken.

#### Analysis and integration

Descriptive statistics from the two surveys were produced using Stata 17. Interviews were professionally transcribed, and integrity checked prior to being imported into NVivo and coded using thematic analysis [22]. Having familiarised themselves with the data by listening to the audio recordings during integrity checking, members of the research team coded the data semantically, based upon what was explicitly stated by participants, and latently, reflecting underlying meaning [23]. The research team met regularly to collapse, combine, and clarify codes, and to identify the themes (shown in Table 3). Three themes were identified during analysis of interviews with healthcare professionals, and six in the analysis of the carer interviews.

**Table 3:** Integration of survey, interview, and workshop findings

	**Healthcare professionals**		**Carers**		**Workshop themes**	**Convergence or divergence**
**Areas for improvement**	**Survey example**	**Interview themes**	**Survey example**	**Interview themes**		
Timely recognition of end of life	54.0% of respondents agreed that their workplace/ward recognised dementia as a terminal condition in a timely manner	1) Prognostication is tricky, and end of life is not always recognised and discussed	40.4% of carers said the person knew they were going to die, 43.9% said no, and 15.8% were not sure	1) Discussions about death usually happen at the end	1) Creating resources for end of life for care professionals to use in different care settings	Convergence
Conversations about end of life	61.9% agreed their workplace/ward had a culture of open discussion about death and dying		When asked if the person who told them they were likely to die broke the news in a sensitive and caring way, 45.6% of carers said this did not apply as they did not know they were dying, or they did not tell them (24.6%). Only 17.5% said yes, and 3.5% said no		2) Having early conversations about end of life	Convergence
Information and support for people with dementia and carers	Free text comment: “More open discussions with patients and families about stage of dementia and preparing for end-of-life care.”	2) People with dementia, family and carers lack information and support	In the last two days of the care recipient’s life, 74.4% of carers agreed they had a supportive relationship with healthcare professionals	2) Caring is challenging 3) Knowledge and understanding of dementia and end of life	3) Increasing education and support available to carers	Overall convergence with some divergence
Person and carer centred care	Free text comment: “More collaborative approach with the staff and family altogether rather than just medical team.”	3) Quality end-of-life care involves managing symptoms, maintaining dignity and comfort care	71.9% of carers said they were involved in decisions about care as much as they wanted to be	4) People’s experiences with dementia differ	4) Improving communication and consultation with carers	Overall convergence with some divergence
Accessing quality, coordinated care	54.4% of respondents agreed if they had a dying relative in hospital, they would feel confident in the excellent quality of care that could be delivered in their workplace/on their ward		64.3% of carers rated the care provided in the last three months of the life of the person they cared for as outstanding, excellent, or good	5) Experiences with services and supports vary 6) Difficulty accessing support	5) Better coordinated services	Overall convergence with some divergence

Narrative integration was carried out by the first author, with all other authors providing critical feedback [24]. The survey, interview, and workshop findings were mapped out, with each given equal priority, resulting in the identification of five areas in which we can improve care for people with dementia at the end of life. Survey, interview, and workshop findings grouped in each of the five areas were assessed to determine whether there was: 1) convergence; 2) overall convergence with some divergence; 3) overall divergence with some convergence, or 4) divergence. See Table 3.

## Results

### Timely recognition of end of life

In the survey of healthcare professionals, around half of respondents agreed (54.0%) that their workplace/ward recognised dementia as a terminal condition in a timely manner. Most agreed (77.3%) that they were confident in their own abilities to recognise when a patient was dying. When asked about timely withdrawal of acute care, only 6.3% of respondents said this always happened, while 84.4% said it happened sometimes, or usually. Less than 10% said rarely or never. A survey respondent commented:

*“[It] appears medically there is no clear guidance to when a patient with dementia is in the terminal stage of their illness and therefore this is not recognised until the terminal (days) phase.” (Nurse, Geriatrics)*

This limited recognition of end of life for people with dementia was also identified in the interviews with healthcare professionals. A nurse specialist spoke about a lack of acknowledgement of end of life from the medical team, saying:

*“In palliative care we see a patient and we go, ‘That’s someone the cleaner could have told you was dying.’…but the medical team looking after them are just going, ‘No we’ve got to keep going, no we’ve got to keep going,’”*

A clinical nurse consultant working in the hospital also highlighted this issue:

*“I had a geriatrician just recently with another patient who I went to talk to them about making the patient palliative care and they said, “Oh, he's not at that stage yet.” And the patient died that night.”*

They went on to explain:

*“It's still out there in the community, that perception that – it's not recognised as a terminal disease. It's frustrating because if somebody had a diagnosis of cancer and was approaching the end of life, people would have no problem whatsoever with withdrawing treatment and putting them on a comfort approach.”*

In the workshop with healthcare professionals, creating resources for end of life for care professionals to use in different care settings was identified as a priority issue, which converged with comments made by healthcare professionals in the survey:

*“Having a uniform screening tool to address when a patient is transitioning to end of life would help identify patients earlier and implement the appropriate care earlier.”*

In these comments, one healthcare professional explained how these tools might help to trigger multidisciplinary meetings when they believe a patient may be dying. This experience of untimely recognition of end of life was also found in the survey and interviews with carers. When carers were asked about awareness of end of life in the survey, 40.4% said that the person knew they were going to die, 43.9% said no, and 15.8% said they were not sure. In their interviews, carers described the end-of-life experience for the person they cared for as happening “quite quickly”, “really quickly”, within hours, days, and weeks.

### Conversations about end of life

In the workshop with healthcare professionals, having early conversations about end of life was identified as a priority issue. In the online survey of healthcare professionals, only 61.9% agreed their workplace/ward had a culture of open discussion about death and dying. Having discussions about death and dying with carers was raised as a particular challenge, with one nurse specialist sharing the following story in an interview:

*“I was invited to speak at the Dementia Carer’s Support Group a few years ago, and these were all carers who – their person, whether it was a spouse or a friend, was already in a residential aged care facility…but when I walked in to say, ‘I’m here to talk about palliative care’, they went, ‘Oh but we don’t need to talk to you yet.’”*

These findings converged with carers’ survey responses. When asked if the person they cared for was told they were likely to die and had this news broken in a sensitive and caring way, most carers said that this did not apply as the person did not know they were dying (45.6%) or they did not tell them (24.6%). Only 17.5% said yes, and 3.5% said no. In their interviews, carers only described conversations about end of life as happening right at the end. A woman who cared for her father said:

*“His lungs were badly damaged, he had pneumonia and we were trying with medications to help the pneumonia and getting better. But really, I think that was just a useless process. I don't really think that was ever going to happen. And it's a bit of a shame that someone didn't have the hard conversation with us from there, just to say, ‘Look, this is really serious. His lungs are quite damaged.’ No one ever really told us that he wasn't coming out of hospital again.”*

This quote illustrates a desire from some carers for open, honest conversations, and avoidance of futile interventions.

### Information and support for people with dementia and carers

The need for more information and support for carers featured in the findings from the healthcare professionals’ survey and interviews. A palliative care nurse specialist working in the hospital explained that there was often misinformation about palliative care:

*“They see us walking with a black sign and a hood over our heads. I think particularly in the hospital, but even when I’ve seen people at home, they almost assume that you’re coming to measure them up for their coffin.”*

A senior occupational therapist working in the community explained:

*“One of my biggest reflections is probably how people access additional support. So how, partners, loved ones, carers, sons and daughters, and so forth, actually get some support when they need it. And they might put out an inkling, they finally put their hand up for some support and there is none.”*

Interviews with carers also highlighted how challenging it is to care for someone with advanced dementia. A woman who cared for a parent said that “It's a hard job, not because of what you're doing, but because it's relentless.” A man who cared for his spouse spoke about his need for information about advanced dementia:

*“It’s hard to describe things when you live with a person that’s dying…they get aggressive and all this kind of business. And it’s hard not to be aggressive with them. Because you don’t know and I’ve never lived with anyone that’s had dementia. So I really didn’t know what I was supposed to do.”*

He also spoke about the lack of support after she died:

*“I got none of that, there was nothing. I was never told that there were any support services or anything like that; there was nothing. Once she was taken out of the hospital that was the end of it.”*

Two sisters caring for their mother spoke about how beneficial it was to have information on how to care for someone with dementia:

*“Mum might say something to me and I'd say that's not right. The geriatrician would say, just agree with her. And I found that, you know, like I think giving advice to the actual carers of how to deal with some of the aspects of dementia would be a really helpful tool.”*

In the workshop with carers, information and support for carers was identified as a priority issue.

There was mild divergence with the findings from the survey of carers, who reported high levels of satisfaction with the information and support provided in the last days of life. Most carers agreed they were kept informed on the person’s condition and care (75.5%), they had enough time to discuss the person’s condition and care (78.6%), they understood the information provided (87.2%), and had a supportive relationship with the healthcare professionals (74.4%). Two thirds of carers said that yes, they were given enough help and support by the healthcare team at the actual time of death (68.4%), but more than one in four said no (28.1%). More than half of carers reported that after the person died, staff dealt with them and their family in a sensitive manner (64.9%), but 15.8% said they did not. More than half of carers said that since the person died, they had not talked to anyone from health and social services, or from a bereavement service, about their feelings about the person’s illness and death. Over half of these carers did not want to anyway (56.1%), but importantly, more than one in four said they would have liked post death support but did not receive it (28.1%).

### Person-and-carer-centred care

In the interviews with both healthcare professionals and carers, the diversity in dementia experiences and need for person-centred care was highlighted: “There's no, really, two similar people that experience it in the same way, is there? And that's why it's so tricky.” (Dietician, working in the community). The need for a carer-centred approach was raised in the healthcare professionals’ survey, with one respondent suggesting the following improvement: “More collaborative approach with the staff and family altogether rather than just medical team.” This was echoed by carers in their workshop, where improving communication and collaboration with carers was identified as a priority issue.

There was mild divergence with the results from the carers’ survey, which suggested most care was person-and-carer-centred. When carers were asked in the survey if the person they cared for was involved in decisions about their care as much as they would have wanted, most said they were not able to be involved (63.2%), one in five said that they were involved as much as they wanted (21.1%), 7.0% said they would have liked to be more involved, and 8.8% said they did not know. When asked if they thought the person had enough choice about where they died, responses were very mixed, with approximately one in four saying yes (22.8%), no (28.1%), not sure (22.8%), or they died suddenly (26.3%). Most carers said they were involved as much as they wanted to be (71.9%), but more than one in four said that they would have liked to be more involved (26.3%). When asked if there were any decisions made about the person’s care that they would not have wanted, around half of carers said no (49.1%), while a quarter said yes (24.6%), and more than a quarter said they were not sure (26.3%).

### Accessing quality, coordinated care

In the interviews, healthcare professionals described quality end-of-life care for people with dementia as involving symptom management, maintaining dignity, and providing comfort care: “Not in distress, pain, agitation. Comfortable.” (Nurse Unit Manager, working in the hospital). A nurse working in hospital, community, and residential aged care settings, described how lack of access to quality, timely care impacts people with dementia, carers and healthcare professionals, resulting in increased carer stress and healthcare professional workload:

*“…a lot of people have been approved for aged care packages at lesser grades so, you know, at level one or a level two, but then they’re really needing a level four, the delay in those, even that in itself, is stressful for carers. So that impacts my ability to care for them because we’re dealing with a household with a lot of stress and carer burnout, that can generally at times, sometimes lead to hospital admission, so increasing hospital admissions for carers with carer stress. It can make it difficult to engage with those carers as well when they’re experiencing carer stress…their retention of information is generally reduced because obviously stress impacts the brain and, so your time is generally required more frequently for repeat things, so things that you may have discussed with them previously you’re having to discuss with them all over again because they’re just not retaining that level of information. So it generally increases the workload and because we work, my nature of work majority being in the community, when our patient or our carers are experiencing carer stress they will contact our service more frequently, their threshold to contact us is generally light or quite low, so it then in turn drives up our own workload.”*

Staffing was highlighted in the interviews by a nurse working in the hospital as being a barrier to providing quality care:

*“I hate to say staffing, I hate to say that, but I think that would really help those areas that the wards that struggle with it, because it’s a common thing I hear in those wards, they’re just overworked, and, I think the care needs are high on those wards, so I feel like those areas could do with more staff but that’s a whole lot of battle.”*

The quality of care provided by a small minority of staff was also raised as an issue by another nurse working in the hospital:

*“It’s a hard area to get people interested in and involved in…the quality of staff generally overall are great, but I suppose it’s a small minority that don’t apply the techniques that we’d like to, I suppose, use more often.”*

These interview findings were concordant with the survey of healthcare professionals, with only 54.4% of respondents agreeing if they had a dying relative in hospital, they would feel confident in the excellent quality of care that could be delivered in their workplace/on their ward. In their interviews, carers also described varying experiences with services and supports. One man who cared for his mother spoke about poor quality care from her GP, saying: “Maybe she’d been with him too long, I don’t know, but in the end it’s just like ‘Here’s the tablets. See you later. Next.’ You know?” Sisters who cared for their mother described their experiences of receiving high quality care, saying their Geriatrician provided information and advice, and facilitated communication with nursing home staff.

**Fig. 1:**
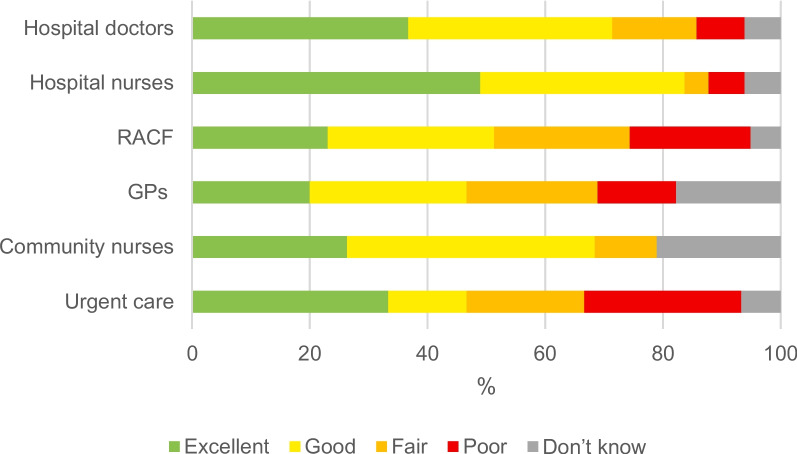
Overall care quality rating for urgent care, community nurses, GPs, RACFs, and nurses and doctors in hospital

However, there was some divergence in the findings from the carer survey. When asked to take all services into account, and rate their care in the last 3 months of their life, more than half of carers said that it was outstanding, excellent, or good (64.3%), around one in five said that it was fair (19.6%), 14.3% said that it was poor, and one carer (1.8%), said that they were not sure. Nurses and doctors in the hospital and community were most likely to be rated excellent or good (83.7%, 71.42%, 68.4%); urgent care, residential aged care facilities (RACFs), and GPs were most likely to be rated fair or poor (46.7%, 43.6%, 35.6%). See Fig. 1. Healthcare professionals and carers in their respective workshops, discussed care coordination as a solution needed to improve coordination and improve perceptions of the quality of care.

## Discussion

This study aimed to identify ways to improve end-of-life care provided to people with dementia and their carers. The mixed-methods findings were integrated into five areas, with results from the five data sets converging, or having overall convergence for ‘Timely recognition of end of life’, ‘Conversations about end of life’, ‘Information and support for people with dementia and carers’, ‘Accessing quality, coordinated care’, and for ‘Person-and-carer-centred care’.

Findings from the surveys and interviews showed that there is a need to improve recognition and communication about the progressive, terminal nature of dementia, as well as changes that may indicate a person is reaching the terminal phase. Resources to support identification and care planning for people with dementia at the end of life was identified by healthcare professionals in the workshops as a solution to these issues. Having early conversations about end of life was another priority area for improvement identified in the study. The findings highlighted the need for a more open culture of discussion of death and dying in healthcare settings and in the community.

The need to better inform and support people with dementia and their carers was raised not only by the carers participating in the study, but also the healthcare professionals. Most carers reported being satisfied with the information and support in the last days of life, perhaps reflecting the increased ability for healthcare professionals to deliver, and people with dementia and carers to receive end-of-life care when death is imminent. The overall findings highlighted how beneficial it is for families to have an understanding of palliative and end-of-life care and information on how to care for someone with dementia in the last 12 months of life. Findings from the interviews with both carers and healthcare professionals made it clear that people’s experiences of dementia differ significantly. Given the multitude of experiences, carers and healthcare professionals agreed on the need for a person-and-carer-centred approach, with improved communication and collaboration between all parties involved.

Integration of the datasets revealed participants’ experiences of providing and receiving both high and low quality end-of-life care. Barriers to providing high quality care, and the impacts of delivering poor quality care were also stressed. This is consistent with Sachs et al., (2004) previous research, which identified symptom management and the stress experienced by family carers as barriers to excellent end-of-life care for people with dementia [25]. Care coordination was a solution offered up by healthcare professionals and carers in their separate workshops. These findings highlight some differences in end-of-life care between “routine” palliative care for those with or without dementia. We discuss these under the following headings:

### Timely prognostication and conversations about end of life

Unlike cancer, where the prognosis can be reasonably inferred from the stage of the disease and the response to therapy, dementia is much more variable [26]. Prognostication is hindered by the fact that an individual may succumb slowly due to the frailty and malnutrition that accompanies dementia, or quickly from an acute event such as aspiration or a fall and hip fracture. This is evidenced in the carers’ survey that less than half of the person with dementia (40.4%) knew that they were going to die. Healthcare professionals in both the survey and workshop identified a uniform screening tool as a priority to help them with recognising transition of person with dementia to end of life. In line with previous literature [25, 27], this study suggests that for people with dementia, it may be worth erring on the early side for referral and documenting conversations around preferences for the end of their lives. Given that the ethos of palliative care is to facilitate best quality of life according to patient preferences [4], it behoves the treating team to initiate these discussions proactively when the dementia is still in its early stages and the patient is able to participate as much as possible in eliciting these preferences.

### Care coordination

Comorbidities have major implications for the care of people with dementia. The most common comorbidities in the last five years of life are cardiometabolic disorders, neurological and cerebrovascular diseases, and musculoskeletal, thyroid, and psychiatric disorders [28]. Therefore, it is often unavoidable that several healthcare professionals are involved in the medical care of the person with dementia, e.g., cardiologist, diabetologist, geriatrician, as well as the GP. An ideal model of care would see that the various healthcare providers are coordinating to meet the needs of person with dementia and their carers at end of life [29], as suggested by both healthcare professionals and carers during workshops in this study. The clinical implication of having multidisciplinary teams and involving carers early on is fundamental in informing and supporting both person with dementia and their carers handling symptoms and behavioural changes, developing goals of care, and shared decision-making. Social workers, in particular, occupy an important role in the multidisciplinary team, linking carers in with services and support groups that provide practical information. Regular and ad hoc meetings that include people with dementia and carers, coordinated by a case manager, is a model which has shown promise [30]. This is of particular importance given that experience with dementia differ vastly. This unified and person-and-carer-centred approach would be particularly important given the difficulty in navigating the aged care support programs (support at home vs residential aged care facilities) let alone juggling the palliative care issues.

### Support for grief and loss

A person’s cognitive changes resulting from dementia can contribute additional challenges for carers as end of life approaches. Ascertaining the person’s wishes, judging symptom burden and comfort level, and engaging in meaningful goodbyes, can be more difficult when someone’s cognition is impaired. This often contributes to more complicated grief and high levels of depression, which is estimated to be experienced by 20% of dementia carers [31]. In the workshop carers outlined the need for more information and support, and the survey found that one in four carers were not receiving the post-death support they wanted. This finding relates to previous studies which have found that bereavement and support are core to models of end-of-life care seeking to improve experiences for people with dementia and their carers in Europe [32], or in community settings [33].

### Strengths and limitations

The study was not resourced to allow for a separate application to the Aboriginal Human Research Ethics Committee, and therefore, following advice from Aboriginal stakeholders, we excluded carers of deceased people who identified as being of Aboriginal or Torres Strait Islander origin. While work has been done to increase capacity in those delivering palliative care services to Aboriginal and Torres Strait Islander peoples [34], future research is needed focussing specifically on care for Aboriginal and Torres Strait Islander people with advanced dementia. Other limitations include potential bias in our respondents: healthcare professionals with a strong interest in palliative care would be more likely to complete the survey or volunteer for an interview, while for carers, the bias could be towards those who either had worse experiences and were eager to share them or had great experiences and were equally eager to share them. This bias is especially acute given the low response rate. Other studies utilising an adapted VOICES-SF report that making phone contact with carers is critical to ensuring a reasonable response rate [35]. For carers, there was 2–7 years between their bereavement and when they participated in the study, and this may impact their recall. A retrospective design such as ours also does not capture the voice of the person with dementia; to capture their beliefs, preferences and choices adequately would require a prospective design where those with dementia were interviewed earlier in the course of their illness, with follow-up to see how congruent their care was with their wishes. The strengths of our study were that the themes were constructed from a synthesis of both healthcare professional and carer perspectives and included mixed methods that combined both surveys and open questions, allowing the greatest freedom to identify themes.

## Conclusions

To address gaps in the literature, this study aimed to integrate multiple perspectives on end-of-life care for people with dementia and carers and to identify clinically relevant areas for improvement. The clinical implications from the identified solutions include that; 1) the heterogeneous and challenging experiences of living with and caring for people with dementia necessitate a multidisciplinary, multifaceted approach to end-of-life care, with early conversations discussing and planning for end of life, and tools to support identification of end-stages, 2) an optimal care journey requires a coordinated response among various health professionals, across the primary, secondary and tertiary care sectors, with people with dementia and their carers at the centre, 3) this person-and-carer-centred approach is essential to improve the communication, collaboration, and support for people with dementia and their carers. These identified clinically relevant areas for improvements provide healthcare professionals and policy makers with guidance for local development of a co-designed model of end-of-life care, which warrants further research.

### Supplementary Information

**Additional file 1. **Healthcare professional survey.

**Additional file 2. **Carer survey.

**Additional file 3. **Healthcare professional interview schedule.

**Additional file 4. **Carer interview schedule.

## Data Availability

The datasets generated and analysed during the current study are not publicly available due ethical restrictions requiring data to not be shared outside of the research team.

## Abbreviations

GP: General Practitioner
REDCap: Research Electronic Data Capture
RACFs: Residential aged care facilities
VOICES-SF: Views of Informal Carers—Evaluation of Services (Short form)
