# Supplementary material for: Improving end-of-life care for people with dementia: a mixed-methods study
Source: BMC Palliat Care. 2024 Jan 30;23:30. doi: 10.1186/s12904-023-01335-w (PMC10825990; doi:10.1186/s12904-023-01335-w)
Supplement: Supplementary file 1 — Additional file 1. Healthcare professional survey. [file 12904_2023_1335_MOESM1_ESM.pdf]

Improving the end of life care journey for people with dementia and their carers

Supplementary file 1: Healthcare professional survey

Improving the end of life care journey for people with dementia and their carers Thank you so much for being interested in the End of Life Dementia Journey project.

This survey is adapted with permission from the Australian Commission on Safety and Quality in Health Care, End-of-Life Care Toolkit: Clinician survey questions.

This survey will take approximately 10 minutes to complete and has three sections:

About you End of life care in your workplace/ward Your role in end of life care. Responses are due back by 16 May.

By completing and returning this survey, you agree to participate in our study. All the information you provide in this survey is anonymous and will be kept confidential.

Please ensure you have read the information statement below before completing this survey.

Participant Information Statement

[Attachment: "Information statement.pdf"]

In order to participate in this survey, you will need to answer 'Yes' to the following questions

---

Do you provide end of life care to people with dementia or their family and friends?

- ◯ Yes
- ◯ No

Do you provide care in hospital, community, or residential aged care facility settings?

- ◯ Yes
- ◯ No

Do you provide care on the Central Coast?

- ◯ Yes
- ◯ No

Section 1: About you

How do you describe your gender?

◯ Man or male
◯ Woman or female
◯ Non-binary
◯ I use a different term
◯ Prefer not to answer

Please specify ______________________

Which country were you born in? ______________________

Clinician cohort

◯ Consultant
◯ General practitioner (GP)
◯ Junior Doctor
◯ Nurse
◯ Allied health professional
◯ Care worker
◯ Community case manager
◯ Other

Please specify ______________________

Years of clinical experience since graduation

◯ 0-1 year
◯ 2-5 years
◯ 6-10 years
◯ >10 years

Major speciality of patients you cared for

◯ Geriatrics
◯ Medical
◯ Surgical
◯ Palliative Care
◯ Rehabilitation
◯ Emergency Medicine / Critical Care
◯ Psychiatry / Mental Health
◯ Other

Please specify ______________________

Place where you give majority of care. Select all that apply.

☐ Hospital ward
☐ Patient's private home
☐ Residential aged care
☐ Private rooms / clinic
☐ Outpatient clinic
☐ Other

Please specify ______________________

Section 2: End of life care in your workplace / ward

The following questions ask about your experiences with end of life care at work. In this project, end of life care is defined as the final 12 months of life.

In answering these questions, consider "in my workplace / on my ward" to be the environment where you provide most care to patients with dementia.

End of life care is done well in my workplace / on my ward

◯ Always
◯ Usually
◯ Sometimes
◯ Rarely
◯ Never

I am confident in my ability to recognise when a patient is dying

◯ Strongly agree
◯ Agree
◯ Neither agree nor disagree
◯ Disagree
◯ Strongly disagree

People in my workplace / ward are very skilled at recognising when a patient is dying

	Strongly agree	Agree	Neither agree nor disagree	Disagree	Strongly disagree	Not applicable
Consultants or GPs	○	○	○	○	○	○
Junior Doctors	○	○	○	○	○	○
Assistant in Nursing or Personal Care Assistant	○	○	○	○	○	○
Enrolled Nurse	○	○	○	○	○	○
Registered Nurse	○	○	○	○	○	○
Nursing Staff with added responsibilities (NUM, CNC, CNS, CNE, Other)	○	○	○	○	○	○
Allied Health Professionals	○	○	○	○	○	○

Decisions about end of life care for patients who are dying are made in a timely manner

	Strongly agree	Agree	Neither agree nor disagree	Disagree	Strongly disagree	Not applicable
Consultants or GPs	◯	◯	◯	◯	◯	◯
Junior Doctors	◯	◯	◯	◯	◯	◯

Dying patients in my workplace / on my ward receive a timely withdrawal of acute treatment

◯ Always
◯ Usually
◯ Sometimes
◯ Rarely
◯ Never

My workplace / ward

	Strongly agree	Agree	Neither agree nor disagree	Disagree	Strongly disagree	Not applicable
Has a culture of open discussion about death and dying	◯	◯	◯	◯	◯	◯
Recognises dementia as a terminal condition in a timely manner	◯	◯	◯	◯	◯	◯
Promotes a palliative approach to care for patients with dementia (i.e. focus on comfort and symptom management)	◯	◯	◯	◯	◯	◯

Please provide comments on your above answers

The majority of end of life care decisions in my workplace / on my ward are made by:

◯ Nurses
◯ Interns / Residents
◯ Junior Registrar
◯ Advanced Trainee Registrar
◯ Consultant
◯ GP
◯ Intensive Care / Medical Emergency Team
◯ Palliative Care Team
◯ Allied Health Professional
◯ Care Managers / Clinical Coordinators
◯ Unsure
◯ The decisions are generally made before arrival in my workplace / on my ward

Please specify discipline

____

Please provide comments on your above answer

Most of the documentation of resuscitation orders in my workplace / on my ward is completed by: Select one answer only

◯ Nurses
◯ Interns / Residents
◯ Junior Registrar
◯ Advanced Trainee Registrar
◯ Consultant
◯ GP
◯ Intensive Care / Medical Emergency Team
◯ Palliative Care Team
◯ Allied Health Professional
◯ Care Managers / Clinical Coordinators
◯ Unsure
◯ The decisions are generally made before arrival in my workplace / on my ward

---

Please specify discipline

____

Please provide comments on your above answer

If I had a dying relative in hospital, I would feel confident in the excellent quality of care that could be delivered in my workplace / on my ward.

◯ Strongly agree
◯ Agree
◯ Neither agree nor disagree
◯ Disagree
◯ Strongly disagree
◯ Not applicable

Please provide comments on your above answer

Within the multidisciplinary team in my workplace, the roles and responsibilities in caring for the person with end-stage dementia are clear.

◯ Strongly agree
◯ Agree
◯ Neither agree nor disagree
◯ Disagree
◯ Strongly disagree
◯ Not applicable

Please provide comments on your above answer

People in my workplace / on my ward are skilled at talking about death and dying with patients and their families

	Strongly agree	Agree	Neither agree nor disagree	Disagree	Strongly disagree	Not applicable
Consultants or GPs	◯	◯	◯	◯	◯	◯
Junior Doctors	◯	◯	◯	◯	◯	◯
Assistant in Nursing or Personal Care Assistant	◯	◯	◯	◯	◯	◯
Enrolled Nurse	◯	◯	◯	◯	◯	◯
Registered Nurse	◯	◯	◯	◯	◯	◯
Nursing staff with added responsibilities (NUM, CNC, CNS, CNE, Other)	◯	◯	◯	◯	◯	◯
Allied Health Professionals	◯	◯	◯	◯	◯	◯

Please provide comments on your above answers

How could end of life planning and care of the dying be improved in your workplace / on your ward?

Section 3: Your role in end of life care

The following questions ask about your thoughts on your professional role in providing end of life care.

It is part of my role to talk to doctors about the care of patients who I think might be dying

◯ Yes
◯ No
◯ Unsure

It is part of my role to talk to patients and their families about death and dying

◯ Yes
◯ No
◯ Unsure

I am confident in my ability to talk to patients and their families about death and dying

◯ Strongly agree
◯ Agree
◯ Neither agree nor disagree
◯ Disagree
◯ Strongly disagree
◯ Not applicable

Please provide comments on your above answers

I have received formal education / training on: Select all the apply

☐ How to recognise when patients are dying
☐ How to care for dying patients
☐ How to communicate with patients and families regarding end of life care
☐ How to communicate with patients who are dying
☐ How to support cultural diversity / cultural preferences regarding death and dying
☐ How to care for patients with end-stage dementia
☐ How to recognise delirium
☐ None of the above

Please provide comments on your above answers

How often does the following occur?

	Always	Usually	Sometimes	Rarely	Never	Not applicable
Being involved in the care of the dying causes me some distress	○	○	○	○	○	○
Being involved in the care of the dying is professionally satisfying	○	○	○	○	○	○
I promote a palliative approach to care for patients with dementia (i.e. focus on comfort and symptom management)	○	○	○	○	○	○
Patients' cultural preferences regarding death and dying are supported	○	○	○	○	○	○
Patients' resuscitation decisions are clearly documented in the notes	○	○	○	○	○	○
The palliative care team are consulted in the care of my dying patients	○	○	○	○	○	○
The palliative care team are called at the right time for dying patients	○	○	○	○	○	○

Please provide comments on your above answers

Closing questions

What do you find most challenging about caring for a person with end-stage dementia?

What do you find most rewarding about caring for a person with end-stage dementia?

Please include any other comments or suggestions related to your experiences with dementia and end of life care.
