# Supplementary material for: Improving end-of-life care for people with dementia: a mixed-methods study
Source: BMC Palliat Care. 2024 Jan 30;23:30. doi: 10.1186/s12904-023-01335-w (PMC10825990; doi:10.1186/s12904-023-01335-w)
Supplement: Supplementary file 2 — Additional file 2. Carer survey. [file 12904_2023_1335_MOESM2_ESM.pdf]

Improving the End of Life Care journey for People with Dementia and their Carers

Supplementary file 2: Carer survey

Improving the End of Life Care journey for People with Dementia and their Carers Thank you for choosing to complete this survey. It will take approximately 30 minutes to complete. There is an option to save your responses and return to complete the survey later if you would like to take a break.

You may find these questions distressing. If so, please seek support. There is a list of bereavement services provided in the Information Statement. They are also listed at the end of the survey.

By completing this survey, you agree to participate in this study. All the information you provide is anonymous and will be kept confidential. Please ensure you have read the Invitation Letter and Information Statement below before completing the survey.

Please note that we must receive your completed survey responses by 4 July 2022 for them to be included in the research.

You are also invited to participate in a one-on-one, in-depth interview about your experiences with caring for someone with dementia at the end of life. A Consent Form will be provided at the end of this survey, should you wish to participate.

[Attachment: "Information statement.pdf"]

To access the survey, please enter your Study ID. Your Study ID is printed on the front page of your survey. __________________________

INTRODUCTION

The first question asks about the circumstances surrounding the death of the person to which you provided care. We use the word 'they' to describe this person.

How long had they been ill before they died?

◯ They were not ill - they died suddenly
◯ Less than 24 hours
◯ One day or more but less than one week
◯ One week or more but less than one month
◯ One month or more but less than six months
◯ Six months or more but less than one year
◯ One year or more

Did they spend any time at home during the last three months of life?

◯ Yes
◯ No, they were in a residential aged care facility for the whole 3 months
◯ No, they were in hospital

CARE AT HOME

These questions are about care at home - not in a residential aged care facility.

When they were at home in the last three months of life, did they get any help at home from any of the services listed below? These may be provided by different organisations, such as the local health district, voluntary organisations, a private agency or social services.

- ☐ A community nurse (a nurse who comes to the house)
- ☐ A specialist nurse (a nurse who visits or telephones to talk and advise on medications and other aspects of care)
- ☐ Any other nurse at home
- ☐ Home care worker, home care aide or home help
- ☐ Social worker
- ☐ Counsellor
- ☐ Spiritual support worker
- ☐ Meals on Wheels or other home-delivered meals
- ☐ Occupational Therapist
- ☐ Specialist Palliative Care service
- ☐ Dementia & Behaviour Support Service (DABSS)
- ☐ Behaviour Assessment & Intervention Service (BASIS)
- ☐ Dementia Australia
- ☐ They did not receive any care
- ☐ Don't know
- ☐ Something else, please write below

Please describe:

When they were at home in the last three months of life, did all these services work well together?

- ◯ Yes, definitely
- ◯ Yes, to some extent
- ◯ No, they did not work well together
- ◯ They did not receive any care
- ◯ Don't know

Overall, in the last three months of care, do you feel that you and your family got as much help and support from health and social services as you needed when caring for them?

- ◯ Yes, we got as much support as we needed
- ◯ Yes, we got some support but not as much as we needed
- ◯ No, although we tried to get more help
- ◯ No, we did not ask for more help
- ◯ We did not need help

During the last three months of their life, while they were at home, how well was their pain relieved?

- ◯ Does not apply- they did not have any pain
- ◯ Completely, all of the time
- ◯ Completely, some of the time
- ◯ Partially
- ◯ Not at all
- ◯ Don't know

URGENT CARE PROVIDED OUT OF HOURS

In the last three months of their life, while they were at home, did they ever need to contact a health professional for something urgent in the evening or at the weekend?

◯ Not at all in the last 3 months
◯ Once or twice
◯ Three or four times
◯ Five times or more
◯ Don't know

The last time this happened, who did they contact, or who was contacted on their behalf?

☐ Their GP or the out of hours number
☐ Health Direct
☐ Community nurses
☐ Specialist Palliative Care nurses
☐ They used their personal alarm pendant
☐ 000
☐ Someone else - please write in the space below

Please describe:

What happened as a result? Were they....

☐ Visited by their GP at home
☐ Visited by another GP at home
☐ Visited by a community nurse at home
☐ Visited by a Specialist Palliative Care nurse at home
☐ Given medical advice over the phone
☐ Given another number to ring to get medical advice
☐ Advised to go to an out of hours GP surgery when it opened
☐ Advised to go to an Emergency Department at a hospital
☐ Advised to call 000
☐ Something else. Please write in the space below

Please describe:

Overall, on this last occasion, do you think the health services responded in the right way?

◯ Yes
◯ No
◯ Not sure

Overall, do you feel that the care they got when they needed care urgently in the evenings or weekends in the last three months of their life was:

◯ Excellent
◯ Good
◯ Fair
◯ Poor
◯ Don't know

COMMUNITY NURSES

Did they receive care from community nurses in the last 3 months of life?

◯ Yes
◯ No

How often did the community nurse visit in the last three months of their life (at the most frequent time)?

◯ More than once a day
◯ Every day
◯ 2-6 times a week
◯ Once a week
◯ 2-3 times a month
◯ Less often
◯ Don't know

How much of the time were they treated with respect and dignity by the community nurses in the last three months of their life?

◯ Always
◯ Most of the time
◯ Some of the time
◯ Never
◯ Don't know

Overall, do you feel that the care they got from the community nurses in the last three months of life was:

◯ Excellent
◯ Good
◯ Fair
◯ Poor
◯ Don't know

CARE FROM THE GP

In the last three months of their life, how often did they see the GP they preferred to see?

◯ Always or almost always
◯ A lot of the time
◯ Some of the time
◯ Never or almost never
◯ They didn't try to see a particular GP
◯ They did not need to see a GP

How much of the time were they treated with respect and dignity by the GPs?

◯ Always
◯ Most of the time
◯ Some of the time
◯ Never
◯ Don't know

Were you able to discuss any worries and fears you may have had about their condition, treatment or tests with the GPs in the last three months of their life?

◯ I had no worries or fears to discuss
◯ Yes, I discussed them as much as I wanted
◯ Yes, I discussed them, but not as much as I wanted
◯ No, although I tried to discuss them
◯ No, I did not try to discuss them

Overall, if the GP visited them at home in the last three months of their life, how easy or difficult was it to get them to visit?

◯ Very easy
◯ Fairly easy
◯ Fairly difficult
◯ Very difficult
◯ They wanted the GPs to visit but they would not visit
◯ Does not apply- the GP did not need to visit
◯ Don't know

Overall, do you feel the care they got from the GP in the last three months of their life was:

◯ Excellent
◯ Good
◯ Fair
◯ Poor
◯ Don't know

RESIDENTIAL AGED CARE FACILITIES (RACFs)

Did they live or stay in a RACF at any time in their last three months of life?

◯ Yes
◯ No
◯ Don't know

How much of the time were they treated with respect and dignity by the staff at the last RACF they stayed in?

◯ Always
◯ Most of the time
◯ Some of the time
◯ Never
◯ Don't know

During the last three months of their life, while they were in the RACF, how well was their pain relieved?

◯ Does not apply; they did not have any pain
◯ Completely, all of the time
◯ Completely, some of the time
◯ Partially
◯ Not at all
◯ Don't know

Overall, do you feel that the care they got from the RACF in the last three months of their life was:

◯ Excellent
◯ Good
◯ Fair
◯ Poor
◯ Don't know

LAST HOSPITAL ADMISSION

Did they live or stay in hospital at any time during their last three months of life?

◯ Yes
◯ No
◯ Don't know

During their last hospital admission, how much of their time were they treated with respect and dignity by the hospital doctors and nurses?

	Always	Most of the time	Some of the time	Never	Don't know
Doctors	◯	◯	◯	◯	◯
Nurses	◯	◯	◯	◯	◯

During their last hospital admission, how well was their pain relieved?

◯ Does not apply, they did not have any pain
◯ Completely, all of the time
◯ Completely, some of the time
◯ Partially
◯ Not at all
◯ Don't know

Did the hospital services work well together with their GP and other services outside of the hospital?

◯ Yes, definitely
◯ Yes, to some extent
◯ No, they did not work well together
◯ Don't know

Overall, do you feel that the care they got from the staff in the hospital on that admission was:

	Excellent	Good	Fair	Poor	Don't know
Doctors	◯	◯	◯	◯	◯
Nurses	◯	◯	◯	◯	◯

EXPERIENCES IN THE LAST TWO DAYS OF LIFE

The next questions are about experiences in the last two days of life.

How much of the time were they treated with dignity and respect in the last two days of their life?

	Always	Most of the time	Some of the time	Never	Don't know
Doctors	○	○	○	○	○
Nurses	○	○	○	○	○

Please look at the following statements and tick the answer box that corresponds most with your opinion about the help they received in the last two days of their life?

	Strongly agree	Agree	Neither agree nor disagree	Disagree	Strongly disagree	Does not apply	Don't know
There was enough help available to meet their personal care needs (such as toileting needs)	○	○	○	○	○	○	○
There was enough help with nursing care, such as giving medicine and helping them find a comfortable position in bed	○	○	○	○	○	○	○
The bed area and surrounding environment had adequate privacy for them	○	○	○	○	○	○	○

As far as you are able to say, how much do you agree about the following statements about the overall level of care given by health and social care professionals to them in the last two days of life?

	Strongly agree	Agree	Neither agree nor disagree	Disagree	Strongly disagree	Was not needed	Not sure
In the last two days of life they had sufficent pain relief	○	○	○	○	○	○	○
In the last two days of life they had support to eat or receive nutrition if they wished	○	○	○	○	○	○	○
In the last two days of life they had support to drink or receive fluid if they wished	○	○	○	○	○	○	○

As far as you are able to say, how much do you agree about the following statements about the overall level of care given by health and social care professionals to them in the last two days of life?

	Strongly agree	Agree	Neither agree nor disagree	Disagree	Strongly disagree	Does not apply	Not sure
In the last two days of life care and attention were given to problems apart from pain, thirst and hunger	○	○	○	○	○	○	○
In the last two days of their life their emotional needs were considered and supported	○	○	○	○	○	○	○
In the last two days of life their spiritual and/or religious needs were considered and supported	○	○	○	○	○	○	○
In the last two days of life efforts were made to make sure they were in the place they most wanted to be cared for	○	○	○	○	○	○	○

Overall, how much do you agree with the following statements about communication between you and health care professionals in the last two days of their life?

	Strongly agree	Agree	Neither agree nor disagree	Disagree	Strongly disagree	Don't know	Not applicable
I/we were kept informed on their condition and care	○	○	○	○	○	○	○
I/we had enough time with staff to discuss their condition and care	○	○	○	○	○	○	○
I/we understood information provided to us	○	○	○	○	○	○	○

How much do you agree with the following statement:

	Strongly agree	Agree	Neither agree nor disagree	Disagree	Strongly disagree	Does not apply	Not sure
In the last two days of their life you had a supportive relationship with the health care professionals?	◯	◯	◯	◯	◯	◯	◯

CIRCUMSTANCES SURROUNDING THEIR DEATH

Did they know they were likely to die?

◯ Yes, certainly
◯ Yes, probably
◯ No, probably not
◯ No, definitely not
◯ Not sure

In your opinion, did the person that told them they were likely to die break the news in a sensistive and caring way?

◯ Yes, definitely
◯ Yes, to some extent
◯ No, not at all
◯ Not sure
◯ Does not apply; they did not know they were dying
◯ Does not apply; they did not tell them they were dying

Were you contacted soon enough to give you time to be with them before they died?

◯ Yes
◯ No
◯ I was already there
◯ It was not clear that they were going to die soon
◯ I couldn't have got there anyway

Where did they die?

◯ At home
◯ In the home of another family member or friend
◯ In a hospital ward
◯ In a hospital emergency department
◯ In a hospital intensive care unit
◯ In a hospice
◯ In a residential aged care facility (RACF)
◯ In an ambulance on the way to a hospital
◯ Somewhere else

Did they ever say where they would like to die?

◯ Yes
◯ No
◯ Not sure

Where did they say they would like to die?

◯ At home
◯ In a hospice
◯ In a hospital
◯ In a residential aged care facility
◯ They said they did not mind where they died
◯ They changed their mind about where they wanted to die
◯ Somewhere else

Did the health care staff have a record of this?

◯ Yes
◯ No
◯ Not sure

Do you think they had enough choice about where they died?

◯ Yes
◯ No
◯ Not sure
◯ They died suddenly

On balance, do you think that they died in the right place?

◯ Yes
◯ No
◯ Not sure

Were you or their family given enough help and support by the healthcare team at the actual time of their death?

◯ Yes, definitely
◯ Yes, to some extent
◯ No, not at all
◯ Don't know

After they died, did staff deal with you or their family in a sensitive manner?

◯ Yes
◯ No
◯ Don't know
◯ Does not apply, I didn't have any contact with the staff

Looking back over the last three months of their life, were they involved in decisions about their care as much as they would have wanted?

◯ They were involved as much as they wanted to be
◯ They would have liked to be more involved
◯ They would have liked to be less involved
◯ They were not able to be involved
◯ Don't know

Looking back over the last three months of their life, were you involved in decisions about their care as much as you would have wanted?

◯ I was involved as much as I wanted to be
◯ I would have liked to be more involved
◯ I would have liked to be less involved
◯ Not sure

Looking back over the last three months of their life, were any decisions made about their care that they would not have wanted?

◯ Yes
◯ No
◯ Not sure

Overall, and taking all services into account, how would you rate their care in the last three months of their life?

◯ Outstanding
◯ Excellent
◯ Good
◯ Fair
◯ Poor
◯ Not sure

Since they died, have you talked to anyone from health and social services, or from a bereavement service, about your feelings about their illness and death?

◯ Yes
◯ No, but I would have liked to
◯ No, but I did not want to anyway
◯ Not sure

INFORMATION ABOUT YOU BOTH

What was your relationship to them? Were you their:

◯ Husband / partner / wife
◯ Son / daughter
◯ Brother / sister
◯ Son-in-law / daughter-in-law
◯ Parent
◯ Other relative
◯ Friend
◯ Neighbour
◯ Someone else

What is your age?

◯ 18-19
◯ 20-29
◯ 30-39
◯ 40-49
◯ 50-59
◯ 60-69
◯ 70-79
◯ 80-89
◯ 90+

How do you describe your gender?

◯ Man or male
◯ Woman or female
◯ Non-binary
◯ I use a different term (please specify)
◯ Prefer not to answer

Please specify:

____

What country were you born in? (please state)

____

What was their age when they died?

◯ 18-19
◯ 20-29
◯ 30-39
◯ 40-49
◯ 50-59
◯ 60-69
◯ 70-79
◯ 80-89
◯ 90+

What was their religion?

◯ No religion
◯ Christian (all denominations)
◯ Buddhist
◯ Hindu
◯ Jewish
◯ Muslim
◯ Sikh
◯ Other

Please specify:

____
