# Supplementary material for: Improving end-of-life care for people with dementia: a mixed-methods study
Source: BMC Palliat Care. 2024 Jan 30;23:30. doi: 10.1186/s12904-023-01335-w (PMC10825990; doi:10.1186/s12904-023-01335-w)
Supplement: Supplementary file 3 — Additional file 3. Healthcare professional interview schedule. [file 12904_2023_1335_MOESM3_ESM.pdf]

Supplementary file 3: Healthcare professional interview schedule

End of life care for people with dementia

Interview schedule – Formal care providers

**Interviewer**	
**Participant name and code**	
**Participant phone number**	
**Date of interview**	
**Length of interview**	
**Mode of interview**	Face-to-face / Phone / Online
**Transcript requested**	Y / N
**Summary of findings requested**	Y / N

Introduction:

Hello [participant], my name is [interviewer], and I am a researcher on the end of life care for people with dementia project. Is it still convenient to do an interview today? The interview will take about 20 minutes.

Thank you for taking the time to talk with me. This project aims to improve the experience of Central Coast residents with dementia and their families towards the end of life - which we define to be the last 12 months of life.

Understandably, this can be upsetting or distressing. If you do not want to answer a particular question or questions, would like to take a break, or stop the interview, please let me know. You do not need to provide any reason for this.

We will be recording this interview. The recording allows us to produce a transcript of the conversation, which will be used during the analysis. These transcripts will be kept secure on password-protected systems.

All the information you provide in this interview is anonymous and will be kept confidential. None of what your share today will be published in a manner that would identify you, the person you were caring for, or the care providers you interacted with.

Consent:

- *Do you still have the copy of the participant information sheet that was sent to you?*
- *Do you have any questions about the project, or this interview?*
- *Do you consent to participating in this interview?*

I am starting the recording now.

Interview questions:

- *What is your age?*
- *What is your gender?*
- *What is your job role?*
- *How long have you been working in dementia care?*
- *Do you work publicly, privately, or both?*
- *What setting do you work in – hospital, community or aged care?*

1. **What do you find most challenging, professionally and personally, in caring for people with dementia and/or their carers in the last year of life?**

Prompts:

- *Are you aware of any barriers to care? If so, how might these barriers be overcome?*
- *Is there anything that you need – as a professional – for providing care?*

2. **What do you believe should be the focus of care for a person with dementia in the last 12 months of life?**

3. **We would like to hear about examples of good practice. Please provide an example, from your experience, of what has worked well in the past for providing end of life care to people with dementia and/or their carers.**

Prompts:

- *This can be a model, component, a way of working, or other.*
- *How important do you think continuity and coordinated care is?*
- *What could "good care" look like?*
  - *For patients, their family and carers*
- *What are important elements need to be considered for providing optimal end of life care? (explore two settings; hospital and community settings)*

4. **We would also like to hear about elements of care that haven't worked so well.**

5. **In your opinion, what should be included in a service model for end of life care for people with dementia and/or their carers in the hospital/community?**

Prompts:

- *Which key people/professionals should be included?*
- *What key activities (component, intervention, other) should be included?*

6. **If you could change one thing about how end of life care is provided – what would that be? Why?**

7. **Is there anything else that you would like to add?**

I'm stopping the recording now.

Closing statement:

Thank you for taking the time to talk with me today. The experiences you have shared will be beneficial for researchers and clinicians to understand the end of life journey for people with dementia and their carers. The information will be used to co-design a model of end of life care for people with dementia on the Central Coast.

We are looking for providers to participate in a workshop to develop this model of care.

- *Would you like to participate in the providers' workshop?*
- *Would you like to receive a copy of the transcript from this interview?*
- *Would you like to receive a summary of the findings from the project?*

I understand that talking about end of life care can be challenging. If you are feeling upset about anything from today's interview, please talk to some you trust, a colleague, your Employee Assistance Program at work or GP, or you can call Lifeline's free phone counselling service on 13 11 14.

If you have any questions about this interview or the project, you can contact the research team at any time using the contact details listed on the participant information sheet.
