# Supplementary material for: Improving end-of-life care for people with dementia: a mixed-methods study
Source: BMC Palliat Care. 2024 Jan 30;23:30. doi: 10.1186/s12904-023-01335-w (PMC10825990; doi:10.1186/s12904-023-01335-w)
Supplement: Supplementary file 4 — Additional file 4. Carer interview schedule. [file 12904_2023_1335_MOESM4_ESM.pdf]

Supplementary file 4: Carer interview schedule

End of life care for people with dementia

Interview schedule – Informal care providers

**Interviewer**	
**Participant name and code**	
**Participant phone number**	
**Date of interview**	
**Length of interview**	
**Mode of interview**	Face-to-face / Phone / Online
**Interested in workshop**	Y / N
**Transcript requested**	Y / N
**Summary of findings requested**	Y / N

Introduction:

Hello [participant], my name is [interviewer], and I am a researcher on the end of life care for people with dementia project. Is it still convenient to do an interview today? The interview will take between 30 minutes and one hour.

Thank you for taking the time to talk to me. In the interview, we will discuss your experiences of caring for a person with dementia and their end of life care, in particular, the last 12 months of life. Understandably, this can be upsetting or distressing. If you do not want to answer a specific question or questions, would like to take a break, or stop the interview, please let me know. You do not need to provide any reason for this.

We will be recording this interview. The recording allows us to produce a transcript of the conversation which will be used during the analysis. These transcripts will be kept secure on password-protected systems.

All the information you provide in this interview is anonymous and will be kept confidential. None of what you share today will be published in a manner that would identify you, the person you were caring for, or the care providers you interacted with.

Consent:

- *Do you still have a copy of the participant information sheet that was sent to you?*
- *Do you have any questions about the project, or this interview?*
- *Do you consent to participating in this interview?*

I am starting the recording now.

Interview questions:

- *What is your age?*
- *What is your gender?*
- *What suburb do you live in?*
- *What was your relationship with the person you cared for with dementia?*
- *How long has it been since the person passed away?*
- *Where were they when they died?*

<u>Experiences of providing care</u>

1. **Can you tell me about your experience of caring for [care recipient]**?

Prompts:

- *When did they receive the dementia diagnosis?*
- *How did you feel at that time?*

2. **Do you feel that you had enough support to provide care?**

Prompts:

- *Can you explain that a bit more?*
- *Was there anything or anyone who helped at that time?*

3. How would you describe [care recipient's] last year of life?

Prompts:

- *When did you first realise something had changed in their health status?*
- *Are there any particular episodes or events that you remember and would like to share?*
- *Did you seek additional assistance (for medical or daily life) in the last year of life?*

<u>Your experiences with healthcare professionals</u>

4. Thinking about [care recipient's] last year of life … What healthcare professionals were involved?

5. What were those relationships or experiences like?

Prompts:

- *GP, hospital staff, community care, aged care, allied health*
- *Did you feel they were approachable, that you could ask questions?*
- *Did anyone discuss what was to come and what to prepare for?*
- *Did the health professionals involved work well together?*

6. Tell me about the last interaction you had with healthcare professionals when [care recipient] was nearing the end of their life.

Prompts:

- *Do you think the health service providers responded in the right way?*
- *Can you explain that a bit more?*
- *Did you feel that [recipient's] choices were supported?*
- *Do you feel that your choices were supported?*

7. Other than healthcare professionals who else, or what other services, supported you and [care recipient] during their last year of life?

Prompts:

- *For example, friends, family, Meals on Wheels, church, carer supports, My Aged Care*
- *How did you find this support?*
- *Did you find them useful?*

<u>Final questions</u>

8. Looking back, were there things that services or health workers could have done differently to make things easier for you?

9. What advice would you give to someone who was providing care towards the end of life?

10. Is there anything else (about care at the end of life) that you would like to add?

I'm stopping the recording now.

<u>Closing statement</u>

Thank you for taking the time to talk to me today. The experiences that you have shared will help researchers and clinicians to understand the end of life journey for people with dementia and their carers on the Central Coast.

This information will also be used to develop an improved model of care for people with dementia at the end of life. We are looking for carers to participate in a workshop to help inform this model of care.

- *Would you like to participate in the carers' workshop?*
- *Would you like to receive a copy of the transcript from this interview?*
- *Would you like to receive a summary of the findings from the project?*

I understand that talking about end of life care can be challenging. If you are feeling upset about anything from today's interview, please talk to some you trust, your GP, or you can call Lifeline's free phone counselling service on 13 11 14.

If you have any questions about this interview or the project, you can contact the research team at any time using the contact details listed on the participant information sheet.
